# Role of centchroman in regression of fibroadenoma: A 2-arm randomized control trial

**DOI:** 10.1016/j.clinsp.2024.100567

**Published:** 2024-12-26

**Authors:** Priyanka Rai, Soniya Nityanand, Amarjot Singh, Sunil Kumar Singh, Neha Singh, Arvind Kumar Singh, Dhananjay Singh

**Affiliations:** a Department of General Surgery, Dr. RMLIMS, Lucknow, Uttar Pradesh, India; b Department of Hematology, SGPGIMS, (VC, King George Medical University), Lucknow, Uttar Pradesh, India; c Department of Radiodiagnosis, Dr. RMLIMS, Lucknow, Uttar Pradesh, India; d Department of Community Medicine, Dr. RMLIMS, Lucknow, Uttar Pradesh, India

**Keywords:** Fibroadenoma, Centchroman, Mastalgia, Breast lumps, HADS, Anxiety

## Abstract

**Background:**

Fibroadenomas are common benign breast lumps that can cause anxiety due to malignancy concerns, and Centchroman, a selective estrogen receptor modulator, has shown promise in reducing their size. This study aimed to evaluate the efficacy of Centchroman in reducing fibroadenoma size, mastalgia, anxiety, and depression in affected patients.

**Methodology:**

A parallel-arm randomized controlled trial was conducted at a tertiary care Breast Clinic with 104 patients aged 18‒45 years having fibroadenomas ≤ 3 cm. Group A received Centchroman (30 mg on alternate days) and Group B received a placebo for 12 weeks. The primary outcome was fibroadenoma volume reduction measured by ultrasound, while secondary outcomes included reductions in mastalgia and improvements in anxiety and depression (HADS scores).

**Results:**

Both groups showed fibroadenoma volume reduction over 12-weeks, with a greater reduction in the intervention group (3.67 ± 1.65 cm^3^ to 2.29 ± 1.04 cm^3^) than the control group (3.12 ± 1.16 cm^3^ to 2.73 ± 0.78 cm^3^), though the difference was not statistically significant (p = 0.342 and p = 0.781). However, 28.8 % of the intervention group experienced over 50 % volume reduction compared to 13.5 % in the control group (p = 0.007). Mastalgia significantly improved in the intervention group (VAS: 5.76 ± 2.13 to 2.24 ± 0.93, p = 0.023), with minimal improvement in controls. Anxiety and depression scores significantly decreased in the intervention group, with anxiety dropping from 9 to 5 and depression from 6 to 4 (p = 0.001).

**Conclusion:**

Centchroman effectively reduces fibroadenoma size especially those with multiple fibroadenoma, mastalgia, and psychological distress in patients with fibroadenoma.

## Highlights

• The findings of the study indicate that Centchroman led to a notable reduction in fibroadenoma size, with a significant proportion of patients experiencing more than 50 % regression in their fibroadenomas.
• Patients experienced significant relief from breast pain (mastalgia), showcasing the efficacy of Centchroman in alleviating associated physical discomfort.
• Centchroman led to a significant improvement in anxiety and depression scores, highlighting its positive impact on the psychological health and overall well-being of patients.

## Introduction

The most frequent complaints among women attending breast clinics are pain and the presence of lumps, which often lead to considerable stress and anxiety due to concerns about malignancy.^1^ Fibroadenomas, while benign, are not true neoplasms but rather considered aberrations in the development and involution of the breast's duct-lobular tissue.^2^ This condition involves hyperplasia of the terminal ductal lobular units, causing the fibroadenoma to gradually increase in size. Most fibroadenomas remain small, typically under 3 cm, but some can grow beyond 5 cm, becoming “giant fibroadenomas”.^3^

These masses usually present as painless, firm, smooth, and non-tender lumps that are well-defined and mobile within the breast tissue. Multiple fibroadenomas may also occur in the same breast or bilaterally. Spontaneous regression of these lesions happens in about 10 %–15 % of cases over a period ranging from 6 to 60 months.^3^ Diagnosing fibroadenomas relies on the “Triple Test”, which includes a clinical examination, imaging, and a tissue biopsy, such as Fine Needle Aspiration Cytology (FNAC) or Core Biopsy.^4^ Estrogen is known to play a crucial role in the development of fibroadenomas, with estrogen receptors being identified in the tissue obtained from these lesions.^5^^,^^6^

Treatment options for fibroadenomas traditionally include observation, hormonal therapy, and surgical excision. Centchroman, a novel non-steroidal Selective Estrogen Receptor Modulator (SERM), was developed by the Central Drug Research Institute in Lucknow, India, during the 1980s. Initially introduced as an oral contraceptive, it has minimal side effects, though it can prolong the menstrual cycle in about 10 % of cases and in certain instances of polycystic ovarian disease.^7^ Centchroman acts as a selective estrogen receptor modulator with a weak agonist effect on the endometrium and a strong antagonist effect on the breast duct lobular epithelium.^8^

The potential role of Centchroman in treating fibroadenomas remains an area of growing interest, as a limited number of studies have suggested its positive effect in causing regression of fibroadenomas.^9^^,^^10^ Therefore, in our study, the authors aim to investigate the impact of Centchroman on fibroadenoma regression, measured by serial volume assessments using sonography, and on anxiety and depression in patients with fibroadenoma.

## Methodology

### Study design and setting

The study was a parallel-arm randomized controlled trial with a waitlist control design, allocating participants in a 1:1 ratio. It was conducted at the weekly Breast Clinic operated by the Department of General Surgery at a tertiary care center for 1-year. This tertiary care institute provided the setting for the trial, targeting patients diagnosed with fibroadenoma measuring 3 cm or less. The objective was to evaluate the efficacy of Centchroman in reducing fibroadenoma size compared to a placebo over a period of 12 weeks.

### Study participants

The participants included patients between the ages of 18 and 45, diagnosed with single or multiple fibroadenomas through the triple assessment method ‒ clinical evaluation, ultrasound, and Fine Needle Aspiration Biopsy (FNAC). FNAC was done in these small fibroadenomas as patients were anxious and depressed because of the lump formation due to the increasing awareness of breast cancer. Fibroadenomas could be present in unilateral or bilateral breasts, with the largest fibroadenoma measuring no more than 3 cm. Exclusion criteria included a past or family history of breast carcinoma, patients with polycystic ovarian disease, cervical hyperplasia, liver disease, or other co-morbidities. Pregnant or lactating women within the first six months post-partum were also excluded. The sample size was calculated to be 104 patients, with 52 participants in each group. The sample size was estimated using the Chi-Square test, considering a 40 % hypothesized response rate for the intervention group and 10 % for the control group, with 80 % power and a 95 % Confidence Interval.

### Randomization and allocation

Randomization for group allocation was conducted using a computer-generated random number table, ensuring that participants were distributed into groups without bias. Block randomization with variable block sizes was utilized to maintain balance between the groups throughout the study. Allocation concealment was achieved through the use of Sequentially Numbered Opaque Envelopes (SNOPES). Participants who met the inclusion and exclusion criteria and consented to participate were assigned to one of the two groups by entering their names on the cover of these envelopes. The treatment groups were coded as Group 1 and Group 2, with the actual treatment assignments concealed in sealed envelopes, preventing any bias in group allocation.

The implementation of the randomization and allocation process was handled by a statistician who was not involved in any other part of the study. This statistician generated the random numbers and prepared the sealed envelopes, ensuring that the study investigators and participants were blinded to group assignments. The code for the treatment groups was kept securely with the statistician in a sealed envelope and was only revealed after the completion of data analysis, maintaining the integrity of the blinding process throughout the study.

### Blinding

The study was designed as a triple-blind study. The study groups A and B were randomly encoded as 1 or 2, with the code hidden from the investigators, patients, and analysts until study completion. Two separate types of 14-tablet blister packs were prepared, each tablet labeled for the day it was to be taken, i.e., every alternate day. The blister packs were encoded 1 or 2 by the pharmacist based on the encoding and dispensed to participants based on group allocation through SNOPES.

### Intervention

Group A: Patients received 30 mg Centchroman on alternate days for 3-months.

Group B: Patients received a placebo (Tab Calcium) on alternate days for 3-months.

### Outcome

• Primary Outcome: The primary outcome was the change in the volume of fibroadenoma measured through ultrasound at 12-weeks compared with the baseline measurement at the first visit.
• Secondary Outcome: The secondary outcome was the effect on mastalgia in patients with fibroadenoma and mastalgia.

Patients were classified as responders and non-responders based on the extent of change in volume:

• Responders: 50 % or more reduction in the volume of the largest fibroadenoma compared with its baseline measurement.
• Non-Responders: Less than 50 % reduction in the volume of the largest fibroadenoma compared with its baseline measurement.

### Hypothesis

• Null Hypothesis: There is no association between the use of Centchroman and response (50 % or more volume reduction of fibroadenoma at 12-weeks follow-up compared to baseline measurement).
• Alternative Hypothesis: There is an association between the use of Centchroman and a response.

### Intervention/project implementation

Patients were randomized into two groups:

• Intervention Arm: After randomization, patients received 30 mg of Tab Centchroman orally on alternate days for 12-weeks. Patients were advised to take the medication at the same time each day, not on an empty stomach, and were informed of possible side effects and reassured accordingly. If patients forgot to take the tablet on time, they were instructed to take it as soon as they remembered and continue with the next day's dose as previously advised.
• Control arm: Patients in the control group received a placebo (250 mg tablet of Calcium) on alternate days for 12-weeks.

Ultrasound was performed at the time of enrollment and after 12-weeks. The VAS score for pain was also recorded. This assessment was done by clinicians not participating in the study. After 12-weeks, patients were given the choice to either continue with medical treatment or undergo surgery.

The Hospital Anxiety and Depression Scale (HADS) was used to calculate anxiety and depression before and 3-months of intervention. It is a self-administered tool designed for patients with comorbid physical illness, containing 14-items: seven for anxiety and seven for depression. Each item is scored from 0 to 3, with a total score range indicating psychological morbidity. A score of 11 or more on either subscale suggests significant anxiety or depression, 8–10 indicates borderline cases, and 0–7 is considered normal. The scale excludes somatic symptoms and has been validated for use in the Indian population for screening anxiety and depression.^11^

### Ethical considerations

CTRI registration number ‒ CTRI/2023/07/055015, Ethical clearance was taken from the institute ethical committee (IEC n° 196/22).

### Data analysis

Data were analyzed using SPSS version 24.0. Descriptive statistics such as mean, standard deviation, frequencies, and percentages were used to present the study results. The Chi-Square test was conducted to test the research hypothesis. Probability (p) was calculated to test statistical significance at the 5 % level.

## Results

52 participants in each group were studied. There was no significant difference between the groups in terms of mean age, with the intervention group having a mean age of 29.89 ± 9.65 years and the control group 31.32 ± 10.21 years (p = 0.843). Marital status was also similar, with 65.4 % married in the intervention group and 63.5 % in the control group (p = 1.00). Parity distribution was comparable between the groups (p = 0.692), and there was no significant difference in BMI categories (p = 0.832). Overall, the groups were similar at baseline (Table 1).

**Table 1:** Baseline characteristics of study participants.

Variables		Intervention Group (n = 52)	Control group (n = 52)	p-value
Mean Age (yrs)	29.89 ± 9.65	31.32 ± 10.21	0.843	
Marital status	Married	34 (65.4 %)	33 (63.5 %)	1.00
	Unmarried	18 (34.6 %)	19 (36.5 %)	
Multiple fibroadenoma	38 (73.1 %)	35 (67.3 %)	0.732	
Parity	Nulliparous	16 (30.8 %)	15 (28.8 %)	
	1‒2	25 (48.1 %)	28 (53.8 %)	0.692
	> 2	11 (21.1 %)	9 (17.4 %)	
BMI	< 18.5 kg/m^2^	7 (13.5 %)	4 (7.6 %)	
	18.5‒24.9 kg/m^2^	31 (59.6 %)	37 (71.2 %)	
	> 24.9 kg/m^2^	14 (26.9 %)	11 (21.2 %)	0.832

The volume of fibroadenoma on ultrasonography decreased in both groups over 12-weeks. In the intervention group, the volume reduced from 3.67 ± 1.65 cm^3^ at the first visit to 1.92 ± 1.04 cm^3^ at 12-weeks (p = 0.042). In the control group, it decreased from 3.12 ± 1.16 cm^3^ to 2.73 ± 0.78 cm^3^ (p = 0.781). However, this reduction was statistically significant in the intervention group and not significant in the control group (Table 2).

**Table 2:** Comparison of the volume of fibroadenoma on ultrasonography at first visit and 12-week follow-up.

Group	Volume of fibroadenoma on USG		p-value
	First visit	12-weeks follow up	
Intervention Group (n = 52)	3.67 ± 1.65	1.92 ± 1.04	0.042
Control group (n = 52)	3.12 ± 1.16	2.73 ± 0.78	0.781

**Fig. 1:**
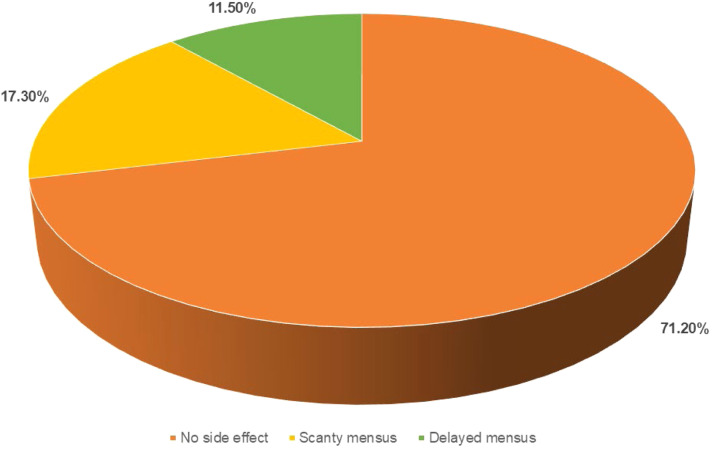
Side effects seen in the intervention group after 12-weeks of Intervention.

In the intervention group, 28.8 % of patients experienced a reduction greater than 50 %, compared to 13.5 % in the control group (p = 0.007) (Table 3). The two groups were compared based on the Visual Analog Scale (VAS) score for mastalgia at the first visit and after 12-weeks of follow-up. In the intervention group, the VAS score significantly decreased from 5.76 ± 2.13 to 2.24 ± 0.93 (p = 0.023). In contrast, the control group showed a reduction from 6.12 ± 1.34 to 4.85 ± 1.46, which was not statistically significant (p = 0.083) (Table 4). In the intervention group, 71.2 % of patients experienced no side effects after 12-weeks of taking the tablet Centchroman, while 17.3 % reported scanty menstruation and 11.5 % had delayed menstruation (Fig. 1).

**Table 3:** Distribution of patients on the basis of reduction in volume > 50 % of fibroadenoma from baseline at 12-weeks follow-up.

Follow-up at 12-weeks		Intervention Group (n = 52)	Control group (n = 52)	p-value
Reduction in volume > 50 % of fibroadenoma from baseline	Yes	15 (28.8 %)	7 (13.5 %)	0.007
	No	37 (71.2 %)	45 (86.5 %)

**Table 4:** Comparison of VAS score for mastalgia at first visit and 12-week follow-up.

Group	VAS score for mastalgia		p-value
	First visit	12 weeks follow-up	
Intervention Group (n = 52)	5.76 ± 2.13	2.24 ± 0.93	0.023
Control group (n = 52)	6.12 ± 1.34	4.85 ± 1.46	0.083

The study showed a significant reduction in both anxiety and depression scores in the intervention group over 12-weeks. The median anxiety score decreased from 9 (IQR: 6‒12) to 5 (IQR: 4‒6) (p = 0.001), and the median depression score decreased from 6 (IQR: 4‒8) to 4 (IQR: 3‒5) (p = 0.001), indicating significant improvements in both anxiety and depression following the intervention (Table 5).

**Table 5:** Comparison of HADS score at first visit and after 12 weeks follow-up.

Group	HADS	First visit	12-weeks follow-up	p-value
Intervention group (n = 52)	Anxiety (Median (IQR)	9 (6‒12)	5 (4‒6)	0.001
	Depression (Median (IQR)	6 (4‒8)	4 (3‒5)	0.001
Control group (n = 52)	Anxiety (Median (IQR)	9 (7‒11)	6(4‒8)	0.074
	Depression (Median (IQR)	7 (4‒10)	5(4‒6)	0.318

## Discussion

The studies on Centchroman (ormeloxifene) for treating fibroadenomas and mastalgia have shown promising results, with many reporting reductions in fibroadenoma size and significant pain relief, though menstrual irregularities were a common side effect. In terms of baseline characteristics, the participants across various studies had a mean age ranging between 27‒32 years. In the study intervention group had a mean age of 29.89 ± 9.65 years and the control group 31.32 ± 10.21 years (p = 0.843). Other studies like Bansal et al. reported a mean age of 32.8 ± 8.35 years,^12^ while Gupta et al. showed mean ages of 27‒31 years across different groups.^13^ Singla et al. included younger participants, with the majority aged between 15‒25 years.^9^ Most studies included a significant proportion of nulliparous women, with Singla et al. reporting 80 % nulliparous patients, and the influence of parity on treatment response was noted in a few studies. Marital status varied across studies.^9^^,^^10^^,^^12^^,^^13^

Regarding fibroadenoma size, several studies noted reductions over time, though the statistical significance varied. Contrary to this study, Shukla et al. observed a reduction in fibroadenoma volume from 4.085 to 3.24 cm^3^ over 12-weeks, though this change was not statistically significant.^14^ In contrast, Tejwani et al. reported significant reductions, similar to the present findings, in the Centchroman group, where the median volume decreased from 2.91 to 0.57 cm^3^ over 24-weeks, while the control group experienced an increase in volume. Singla et al. found that 66.66 % of their participants had a decrease in fibroadenoma size, with 30 % achieving complete resolution.^10^ Similarly, Brahmachari et al. reported that 34 % of patients had a partial reduction, while 46 % experienced complete regression of fibroadenomas. Bansal et al. also showed consistent improvements in nodularity grades, with 92.6 % of patients showing lower nodularity grades by the end of six months,^15^ while Gupta et al. reported an 80 % reduction in fibroadenoma size in their study.^13^ Dhar et al. found that 41 % of fibroadenomas had disappeared entirely after 12-weeks of therapy, with 24 % showing some reduction in size.^16^

In terms of mastalgia (breast pain), significant pain relief was reported in multiple studies which was in concordance with the present results. Dhar et al. showed that all participants were pain-free by the 12-week mark and patients who had recurrences responded well to continued therapy.^16^ Tejwani et al. reported that 92 % of mastalgia patients experienced pain relief with Ormeloxifene.^10^ Similarly, Singla et al. highlighted that fibroadenoma patients with mastalgia showed substantial pain relief after therapy.^9^ Bansal et al. documented a continuous decrease in pain levels, from a mean pain score of 5.8 at the start of treatment to 0.86 by the end of the six-month follow-up.^12^ Girish TU et al. reported that 40 % of patients with mastalgia had no pain by the end of their treatment.^17^

Side effects, particularly menstrual irregularities similar to this study, were common across studies. Tejwani et al. noted significant menstrual changes, with 22.5 % of patients reporting abnormalities.^10^ Amenorrhea (absence of menstruation) was noted in a small percentage of patients in Singla et al., though normal cycles resumed after stopping the medication.^9^ Other side effects were generally mild; for instance, Dhar et al. reported a case of an allergic rash, though it did not require study withdrawal.^16^ Bansal et al. mentioned oligomenorrhea (scanty menses) in a few patients and mild headaches, but no major side effects were observed.^12^ Shukla et al. similarly reported scanty menstruation in 17.3 % of participants and delayed menstruation in 11.5 %.^14^

In treating fibroadenoma and mastalgia, studies have commonly used a dosage of 30 mg on alternate days for a period of 3 months. This dosage has been effective in reducing the size of fibroadenomas and alleviating breast pain in patients with mastalgia. Centchroman has been effective in reducing the size of both single and multiple fibroadenomas, though the authors found that multiple fibroadenomas had better response. Multiple fibroadenomas may experience a partial reduction, with occasional complete regression of a few fibroadenomas, depending on individual patient characteristics and the treatment duration.^18^^,^^19^ However studies have shown to have complete improvement in many single fibroadenoma cases.

The intervention group showed a significant reduction in both anxiety and depression scores over 12-weeks, with median anxiety scores decreasing from 9 to 5 (p = 0.001) and depression scores from 6 to 4 (p = 0.001) in the present study. However, the control group showed a reduction in Anxiety and depression scores but not significant (p > 0.05). In this study, multiple fibroadenoma patients reported a better reduction in HADS score. Similarly, Srivastava V et al. reported that on comparing anxiety and depression using the HADS score, there was a significant difference in both anxiety and depression levels between cases and controls (p < 0.001 for both). After 3-months of follow-up, there was a significant improvement in anxiety and depression scores (p < 0.001 for both). The HADS anxiety score showed significant improvement in patients with diseases other than fibroadenoma, while the HADS depression score showed significant improvement in cases of fibroadenoma, other breast conditions, breast lump, and mastalgia (p = 0.040, p = 0.005, p < 0.001, and p = 0.025, respectively).^20^

Psychosocial stress, severe depression, and Benign Breast Disease (BBD) may be linked through several pathways. Depressed women often have heightened concerns about their health, leading to more frequent self-breast exams, increased medical consultations, and greater sensitivity to minor breast symptoms. Additionally, stress-induced hormone imbalances, particularly increased cortisol levels, may also play a role by affecting other hormone systems, including sex hormones.^21^ This makes it important to assess Anxiety and depression in such patients.

The limitations of the study include that the study was conducted at a single tertiary care center, which may introduce site-specific biases and may not fully represent the wider population affected by fibroadenoma. Additionally, the follow-up period of 12 weeks may not be sufficient to observe the long-term effects of Centchroman on fibroadenoma regression or recurrence, as spontaneous regression of fibroadenomas can take several months to years. The reliance on ultrasound for measuring fibroadenoma size, while effective, may also introduce some measurement variability. Finally, participants were aware that they were part of a clinical study, which could lead to a placebo effect, especially in the control group receiving calcium tablets. The authors did not further follow-up the patients after cessation of the drug whether the fibroadenomas increased in size or were the same. Future studies with larger, more diverse populations and longer follow-up periods could help address these limitations.

In conclusion, Centchroman showed positive results in reducing fibroadenoma size and providing pain relief in mastalgia patients, with few major side effects. While menstrual irregularities were a common side effect, most patients experienced normal cycles after discontinuation of therapy. Overall, Centchroman (ormeloxifene) appeared to be an effective and well-tolerated option for treating fibroadenomas and mastalgia.

## Declaration of competing interest

The authors declare no conflicts of interest.
